# Cognitive style, cortical stimulation, and the conversion hypothesis

**DOI:** 10.3389/fnhum.2014.00015

**Published:** 2014-01-29

**Authors:** David J. M. Kraemer, Roy H. Hamilton, Samuel B. Messing, Jennifer H. DeSantis, Sharon L. Thompson-Schill

**Affiliations:** ^1^Department of Psychology, University of PennsylvaniaPhiladelphia, PA, USA; ^2^Department of Neurology, University of PennsylvaniaPhiladelphia, PA, USA

**Keywords:** cognitive styles, rTMS, conversion hypothesis, verbalizer, fMRI

## Abstract

What does it mean to have a “verbal cognitive style?” We adopt the view that a cognitive style represents a cognitive *strategy*, and we posit the *conversion hypothesis* – the notion that individuals with a proclivity for the verbal cognitive style tend to code nonverbal information into the verbal domain. Here we used repetitive transcranial magnetic stimulation (rTMS) to disrupt this hypothesized verbal conversion strategy. Following our previous research implicating left supramarginal gyrus (SMG) in the verbal cognitive style, we used an fMRI paradigm to localize left SMG activity for each subject, then these functional peaks became rTMS targets. Left SMG stimulation impaired performance during a task requiring conversion from pictures to verbal labels. The magnitude of this effect was predicted by individuals’ level of verbal cognitive style, supporting the hypothesized role of left SMG in the verbal labeling strategy, and more generally supporting the conversion hypothesis for cognitive styles.

## INTRODUCTION

Across various types of situations, individuals often report using either mental imagery or internal verbalizations (or both) in their thought processes. Cognitive styles are thought to represent some of the consistencies in the ways that individuals process information, influencing a range of processes from perception to cognitive control (Witkin et al., 1977; Kozhevnikov, 2007). For example, individuals can readily report their degree of preference for thinking in the verbal modality, such as using spoken or written words to understand a concept. Although an individual’s verbal/visual cognitive style profile appears to be consistently reported across self-report measures (Paivio and Harshman, 1983; Kirby et al., 1988; Massa and Mayer, 2006), and although decades of research have been devoted to the topic (for a review, see Kozhevnikov, 2007), an explicit characterization of what these preferences indicate about cognitive and neural mechanisms remains elusive. Therefore, much debate persists about the precise definition, and utility, of the concept of cognitive style.

One prominent hypothesis regarding the relationship between verbal/visual cognitive styles and learning has been termed the “meshing” hypothesis (Pashler et al., 2008). Under this hypothesis, information presented in a preferred modality (e.g., providing written text for individuals who self-designate as verbal learners) will be better comprehended and better remembered than information presented in a non-preferred modality (e.g., displaying pictures to verbal learners). As Pashler et al. (2008) pointed out, there is no study to date that clearly demonstrates the crossover interaction that would be predicted by this hypothesis (and only a few that were even designed to be able to find one if it were present). That is, it has not been shown that individuals who deem themselves verbal learners actually learn better with words than with pictures, nor that visual learners learn better from pictures than words. This conclusion leads to three possibilities regarding cognitive styles: (1) cognitive styles do not correlate with learning or memory, (2) cognitive styles do correlate with learning or memory, but not in the way that would be predicted by the meshing hypothesis, or (3) the meshing hypothesis is accurate, but the right studies have not been conducted to conclusively demonstrate the predicted effects. The present investigation is aimed at exploring the second possibility – namely that another hypothesis better explains the relationship between cognitive styles and cognition.

**FIGURE 1:**
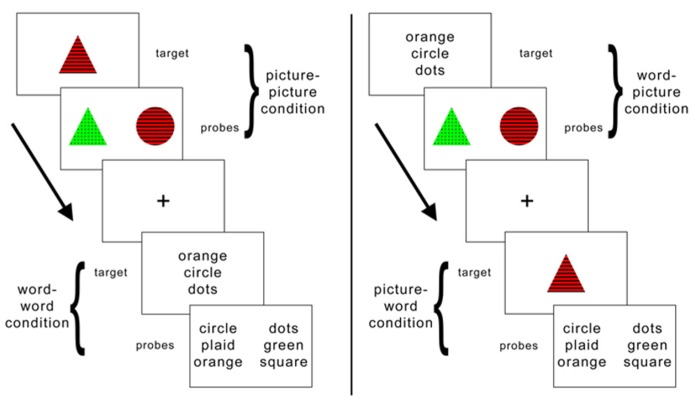
**Schematic illustration of task conditions.** The similarity judgment requires memory and comparison of a target item to two subsequently presented probe items. On half of the trials the target item is presented as a picture and on the other half it is presented as a set of words that name equivalent features. Likewise, the probes are presented as words on half of the trials and as pictures on the other half.

We proposed a hypothesis, termed here the “conversion hypothesis,” on the basis of an interesting effect of cognitive style on fMRI activity during a working memory task (Kraemer et al., 2009). Specifically, we identified brain activity in a region of left supramarginal gyrus (SMG) that, across subjects, responded more to a set of objects represented with words than to a corresponding set of pictorial stimuli (see **Figure 1**). Notably, in a task condition involving the *visual* stimuli alone activity in this *verbal* region correlated with individuals’ ratings on the *verbal* cognitive style. This finding is consistent with the interpretation that subjects who were more likely to engage in verbal thought were activating a linguistic representation of the pictorial stimuli (e.g., a verbal label) in order to complete the task. Thus, in contrast to the meshing hypothesis (Pashler et al., 2008) in which the *input* modality of the stimulus interacts with cognitive style to determine the outcome of behavior; here we see evidence that the *internal* representation of a stimulus is what correlates with cognitive style. In the present study, we explore one consequence of this strategy: if, under normal circumstances, an individual relies on this self-generated representation, what happens to performance when we disrupt the conversion process?

One method often used to disrupt verbal processing is articulatory suppression (e.g., Murray, 1968; Baddeley et al., 1975; Baldo et al., 2005). Despite its frequent usage, however, there is disagreement as to what stage of verbal processing is affected by articulatory suppression (for a review, see Besner, 1987). For example, studies using visually presented stimuli have demonstrated that in several conditions phonological recoding and lexical access can occur during articulatory suppression without any sign of interference (Barron and Baron, 1977; Besner et al., 1981; Baddeley and Lewis, 1981; Besner and Davelaar, 1982; Mitterer, 1982; Bryant and Bradley, 1983; Kimura and Bryant, 1983). In the context of the task used by Kraemer et al. (2009) it is reasonable to conceive that the conversion process associated with the verbal cognitive style is of a similar nature and therefore may not be impeded by articulatory suppression. However, instead of relying on behavioral measures to disrupt this processing, we can leverage the fMRI evidence that implicates a specific brain region in verbal processing of visual stimuli for the verbal cognitive style. Therefore, in the present experiment we use repetitive transcranial stimulation (rTMS) to directly disrupt the activity of this brain region – localized for each participant – and test our hypothesis regarding the verbal cognitive style.

The critical advantage of the verbal cognitive style versus the visual cognitive style under the meshing hypothesis would be in conditions where words are the input, as this meshes the preferred presentation format with the individual’s cognitive style. As mentioned above, when reviewing the extant literature, Pashler et al. (2008), found no support for this hypothesis. In contrast, the conversion hypothesis predicts that the more verbal individuals are more likely to use a verbal processing strategy during encoding irrespective of presentation format. Previous research has demonstrated a mnemonic advantage when both a verbal and a visual code are processed simultaneously (Paivio, 1965, 1979). Thus, the critical advantage of the verbal cognitive style versus the visual cognitive style under the conversion hypothesis would be in conditions where pictures are the input, as in these conditions the individuals who use a verbal strategy would have both a visual code (resulting from the memory of the stimulus) and verbal code (resulting from the verbal label the individual provides at encoding). However, the potential to observe these individual differences is likely to depend on task demands. For example, using the same paradigm as the current experiment, our previous study found no difference in task performance based on cognitive style (Kraemer et al., 2009). Here, using rTMS, we predict that disrupting the verbal strategy will differentially impair task performance for some individuals, to the degree that those participants typically rely on the verbal strategy (represented by their verbal cognitive style score). Thus, in the context of this experiment which uses a two (target: pictures or word) × two (probe: pictures or words) design, these two hypotheses result in the following predictions when rTMS is used to disrupt activity in a brain region (left SMG) that is associated with the verbal cognitive style. The meshing prediction is that during the critical rTMS condition (versus the control site stimulation condition), a decline in performance correlating with verbal cognitive style should be observed for conditions that use words as the input (word–word and word–picture). The conversion prediction is that during the critical rTMS condition (versus the control site stimulation condition), a decline in performance correlating with verbal cognitive style should be observed for conditions that use pictures as the input (picture–picture and picture–word). Moreover, we predict that the strongest effect of rTMS should occur in the picture–word condition, as this condition mirrors the hypothesized verbal conversion – namely, labeling a picture using words and then relying on the labels for further processing. The magnitude of this effect should be predicted by the degree to which individuals are likely to rely on the verbal processing method – i.e., the decline in performance due to rTMS should correlate with verbal cognitive style. As the visual approach should be adequate to complete the task despite disruption of left SMG function, no decline in performance should be observed that correlates with the visual cognitive style.

## MATERIALS AND METHODS

### PARTICIPANTS

We recruited 21 participants (10 male; aged 18–31 years, *M *= 23.50) from the Philadelphia community, most of whom were students or employees of the University of Pennsylvania or the Hospital of the University of Pennsylvania. One participant was excluded due to failure of the functional localizer to identify a peak in LSMG. Participants were paid as compensation for their involvement in this study. All subjects voluntarily agreed to participate, compliant with the informed consent procedures of the Institutional Review Board of the University of Pennsylvania. All subjects reported strong right-hand dominance and no history of seizure or other neurological impairment or injury.

### SIMILARITY JUDGMENT TASK

The behavioral task used throughout this experiment is the same as we used in a previous study (Kraemer et al., 2009; **Figure 1**). Each item consisted of either a picture (e.g., a red triangle with stripes) or three words that named a color, shape, and pattern (e.g., red, triangle, stripes). There were five possible attributes for each of the item features. On each trial, participants viewed a target item (either word set or picture) in the center of the screen for 1500 ms. Next two probe items (either two pictures or two sets of words) appeared for 3500 ms before disappearing. As targets and probes could be presented either as pictures or as words, there were four within-subject trial conditions: picture–picture, word–word, picture–word, and word–picture. Participants completed 38 trials of each of these four conditions during each of two sessions. No three-item set (target and two probes) was ever repeated within or across condition type.

When the probe items appeared, participants pressed a button to indicate which of the two probes was more similar to the previous target. Participants had 3500 ms to respond before the probes disappeared. The probes were then immediately replaced by a central fixation cross for 1 s. The placement (left or right) of the correct answer was counterbalanced across all trials. Correct probes contained two of the three features in common with the target; incorrect items only contained one feature in common. This rule was not explicitly stated to participants; feedback was given on several practice trials, and all participants demonstrated proficiency at the task before the start of the first run.

### COGNITIVE STYLE QUESTIONNAIRES

#### Verbalizer/visualizer questionnaire

Subjects completed a computerized version of the Verbalizer/Visualizer Questionnaire (VVQ; Kirby et al., 1988), which assesses the degree to which an individual employs visual and verbal reasoning in common tasks and situations. The VVQ, following modifications developed by Kirby et al. (1988), comprises 10 statements that relate to a verbal reasoning style (e.g., “I enjoy doing work that requires the use of words.”) and 10 that relate to a visual reasoning style (e.g., “I find illustrations or diagrams help me when I’m reading.”). Participants rated each statement on a discrete five-point scale from strongly disagree (1) to strongly agree (5). Half of the questions on each dimension were reverse scored. Average scores for each dimension were computed for each subject, and possible scores ranged from 1 to 5. The questionnaire was presented on a computer using E-Prime software (Psychology Software Tools, Sharpsburg, PA, USA).

#### Object-spatial imagery and verbal questionnaire

The Object-Spatial Imagery and Verbal Questionnaire (OSIVQ; Blazhenkova and Kozhevnikov, 2008) is a 45-item self-report questionnaire that is divided into three separate dimensions, two of which are visual and one of which is verbal: (1) Object imagery cognitive style, (2) Spatial visualization cognitive style, (3) Verbal cognitive style. Subjects responded to each statement using a discrete 5-point scale (1 = strongly disagree, 5 = strongly agree). Four questions were reverse scored (Blazhenkova and Kozhevnikov, 2008). Average scores for each dimension were computed for each participant. Possible scores range from 1 to 5 for each dimension.

#### Cognitive style factor scores

Averaging the verbal dimensions from both cognitive style questionnaires generated a verbal cognitive style factor score (1–5, 5 = more verbal). Likewise, averaging the visual dimensions from both measures generated a visual cognitive style factor score (1–5, 5 = more verbal). These averaged scores were used for all analyses.

### CORTICAL STIMULATION

To administer rTMS, we used a 70 mm diameter figure-8 coil connected to a Magstim Rapid Transcranial Magnetic stimulator (Magstim, Whitland, UK). We completed processing of the high-resolution structural MRI scan using AFNI (Cox, 1996) prior to importing the images into Brainsight (Rogue Research, Montreal, QC, Canada). We then used Brainsight to co-register the structural scan with the TMS instrumentation. We calculated the resting motor threshold by stimulating the left-hemisphere hand area of motor cortex using single pulses of TMS and visually observing for any movements of the digits or wrist of the right hand (Pridmore et al., 1998). Starting stimulation at supra-threshold intensity and lowering the intensity by steps of 1–2%, we recorded the motor threshold for a participant as the intensity at which we observed hand movement on three out of five stimulation attempts (Balslev et al., 2007). Following parameters that have previously been shown to be effective at depressing cortex excitability for periods lasting at least 15 m post-stimulation (Chen et al., 1997), we applied low frequency (1 Hz) offline rTMS for a period of 15 m (900 pulses) at a stimulus intensity equal to 110% of motor threshold.

### PROCEDURE

**FIGURE 2:**
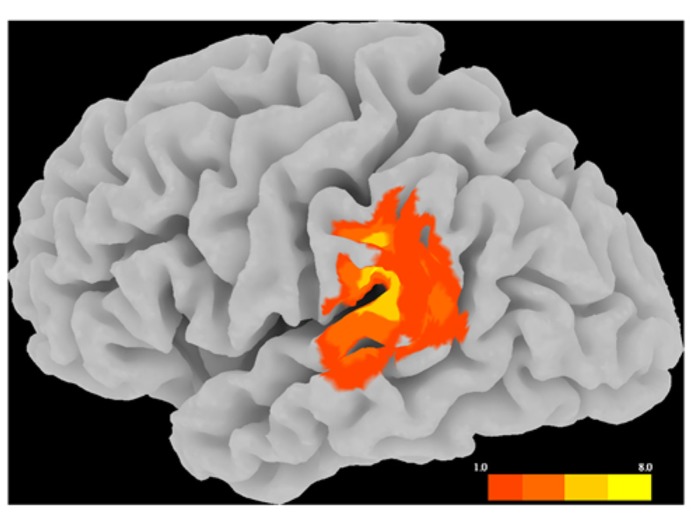
**Distribution of rTMS sites and estimated spread of rTMS effects, displayed as a topographical histogram on a representative brain in standardized space.** The color scale indicates the number of participants estimated to have received stimulation at each voxel.

Participation involved three experimental sessions. In the first session, participants completed the similarity judgment task from our previous study (Kraemer et al., 2009; **Figure 1**) while undergoing functional scanning in order to localize task-specific SMG activity for each participant (**Figure 2**). Following this session the functional data were analyzed as in our previous experiment (Kraemer et al., 2009), specifically by contrasting the activity during the word–word versus the picture–picture conditions. From this contrast the peak activity in left SMG was identified for each subject. In the second and third sessions participants received rTMS and then completed the same behavioral task. We used offline low frequency repetitive TMS (rTMS; for a review of the effects of rTMS and its putative mechanisms, see ref. (Post et al., 1999) to disrupt activity in this region prior to administering the task again. In one session the target of rTMS was the left SMG coordinate identified by the functional localizer. In the other session the target of rTMS was the vertex, used here as a control site. Order of session was counterbalanced across subjects. Verbalizer scores were significantly higher for the group that received vertex stimulation in their first session (M_SMG__→__VTX_ = 3.07, M_VTX__→__SMG_ = 3.73, *t* = 2.09,* p* = 0.03). Because we might expect improvement in task performance upon repeated exposure to the task (i.e., for later versus earlier task sessions), this confound could only work against finding evidence in support of our hypothesis. Therefore, if it has any impact on the present results, it is that we may be underestimating the effect size of our main finding – that performance is differentially impaired by TMS to peri-Sylvian regions as a function of verbal cognitive style. Visualizer scores did not differ between counterbalancing groups (M_SMG__→__VTX_ = 3.24, M_VTX__→__SMG_ = 3.32, *t* = 0.69,* p *= 0.75). The behavioral task was completed (using E-Prime; Psychology Software Tools, Pittsburgh, PA, USA) immediately following application of rTMS and lasted approximately 12 m. More specifically, at each rTMS session, the task was presented in two blocks of 76 stimuli each, with the condition order varying on a trial-by-trial basis in pseudorandom fashion. In the third session, following the completion of the task, the VVQ and OSIVQ were administered and the participants were debriefed regarding the aims of the experiment.

## RESULTS

### LOCATION OF rTMS SITES

**Figure 2** illustrates the variation in SMG rTMS sites via a topographical histogram showing the extent of overlap in stimulation sites between subjects. Rather than display each site as a single point in cortical space, this method represents each site as a spherical ROI (*r* = 8 mm) centered on the peak SMG voxel from the critical fMRI contrast between the picture–picture versus word–word conditions. This radius was chosen to illustrate the likely extent of rTMS effects on cortex, consistent with the estimate that using a standard figure-8 coil at an intensity of less than 120% of motor threshold is likely to stimulate cortex at a depth of up to roughly 1.5 cm (Roth et al., 2007). As clearly seen in **Figure 2**, there is a wide range of variation of rTMS stimulation within left SMG. Additionally, stimulation likely affects nearby peri-Sylvian regions as well, such as superior temporal gyrus (STG).

### TRANSCRANIAL MAGNETIC STIMULATION RESULTS

**Table 1** reports the group-level performance on the behavioral task for each rTMS session. In order to test the overall effects of rTMS on task performance, we conducted a four (task condition: picture–picture, word–word, picture–word, word–picture) × 2 (stimulation site: LSMG, vertex) repeated measures ANOVA using proportion of items correct as a measure of accuracy. Results showed that neither the main effect of stimulation site [*F*(1,19) = 0.87, *p* = 0.36] nor the site*condition interaction [*F*(3,57) = 1.38, *p* = 0.26] had a significant effect on accuracy. There was a significant main effect of task condition on accuracy [*F*(3,57) = 44.23, *p *< 0.0001]. As shown in **Table 1**, for both vertex and SMG sessions the highest mean accuracy was for the picture–picture condition, followed by word–picture, then word–word, then picture–word. *Post hoc* tests confirmed that all pairwise combinations of conditions differed significantly from each other in terms of accuracy (all *t* values ≥ 3.14, all *p *values ≤ 0.005). We also analyzed median response times in the same manner. These results also showed no main effect of stimulation site on response time [*F*(1,19) = 0.30, *p* = 0.59] nor a site*condition interaction for response time [*F*(3,57) = 0.44, *p* = 0.73]. There was a significant main effect of task condition on response time [*F*(3,57) = 187.35, *p *< 0.0001]. As with accuracy, the (ascending) order of conditions in terms of median response time was picture–picture (*M* = 787.31, SD = 194.14), word–picture (*M* = 1253.65, SD = 373.77), word–word (*M* = 1783.43, SD = 324.09), picture–word (*M* = 1847.35, SD = 324.39). *Post hoc* tests confirmed that all pairwise combinations of conditions differed significantly from each other in terms of median response time (all *t* values ≥ 2.95, all *p *values ≤ 0.008). Therefore both accuracy and response time indicate that picture–picture is the easiest condition, followed by word–picture, then word–word, then picture–word.

**Table 1:** Mean accuracy (and standard deviation) for each task condition and each rTMS session.

Condition	Left SMG	Vertex
Picture–picture	0.99 (0.02)	0.97 (0.03)
Picture–word	0.86 (0.07)	0.87 (0.09)
Word–picture	0.94 (0.05)	0.92 (0.06)
Word–word	0.91 (0.07)	0.90 (0.06)
Average	0.93 (0.04)	0.92 (0.05)

**FIGURE 3:**
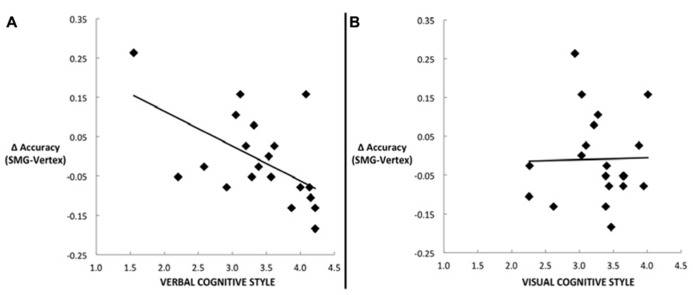
**Scatter plots depicting the correlation between the decline in task performance for the picture–word condition as a result of left SMG rTMS and (A)** the verbal cognitive style (*r* = -0.56, *p *= 0.01), or (**B)** the visual cognitive style (*r* = 0.02, *p *= 0.93).

Our main hypothesis concerned individual differences in the effect of disruption of left SMG function due to rTMS. Specifically, we hypothesized that the verbal cognitive style would correlate with the rTMS-induced decline in performance, specifically for the conditions in which a picture target appeared first (picture–word and picture–picture). **Table 2** reports the Pearson correlations by style and by task condition. For each cognitive style dimension (verbal, visual, and the verbal–visual difference score), a Bonferroni correction was applied based on the number of correlations (i.e., with each of the four task conditions). The one correlation to survive this correction was between the verbal cognitive style and the change in accuracy in the picture–word condition (*r* = -0.56, *p *= 0.01). **Figure 3A** shows a scatter plot of this correlation, which was hypothesized to reflect the verbal labeling strategy, and therefore predicted to show the strongest effect of rTMS correlating with verbal cognitive style. To examine the possibility that an individual data point or small set of data points may be driving this correlation, we used two different measures of influence: studentized residuals and leverage values. One data point was found to have a studentized residual value greater than 2 [*t*(17) = 2.96]. Excluding only this case, the resulting correlation between the verbal cognitive style and the change in accuracy in the picture–word condition was *r* = -0.69, *p* = 0.001. Separately, two other data points were found to have leverage values greater than twice the average value. Excluding only these two cases, the resulting correlation between the verbal cognitive style and the change in accuracy in the picture–word condition was *r* = -0.43, *p* = 0.07. Thus no individual data point or pair of data points significantly alters the pattern of results in terms of effect size (although in the latter case the correlation is no longer significant). **Figure 3B** shows the lack of correlation between the visual cognitive style and the effect of left SMG rTMS on accuracy in the picture–word condition (*r* = 0.02, *p *= 0.93). This is an important negative control, as the task approach associated with the visual cognitive style is not hypothesized to rely on the function of left SMG. This result, therefore, is consistent with our hypothesis. The difference between these correlations is marginally significant [*t*(17) = 1.94, *p* = 0.07], based on Williams’s test (i.e., Steiger’s preferred method).

**Table 2:** Pearson correlations between cognitive style and accuracy difference scores (SMG session - Vertex session) for each task condition.

Condition	Verbal cognitive style	Visual cognitive style	Verbal–visual cognitive style
Picture–picture	-0.38	-0.07	-0.26
Picture–word	-0.56*	0.02	-0.46
Word–picture	-0.27	0.07	-0.26
Word–word	-0.23	-0.05	-0.15

*p < 0.05, corrected.

**FIGURE 4:**
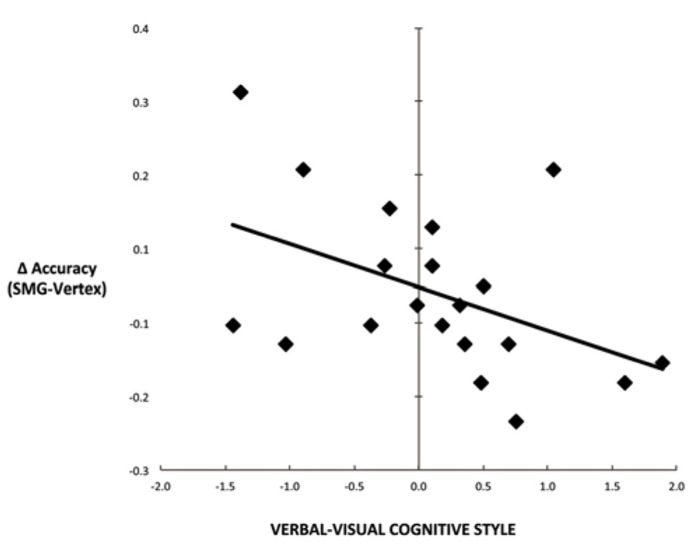
**Scatter plot depicting the correlation between cognitive style difference score (verbal–visual) and the decline in task performance for the picture–word condition as a result of left SMG rTMS (*r* = -0.46, *p *= 0.04)**.

**FIGURE 5:**
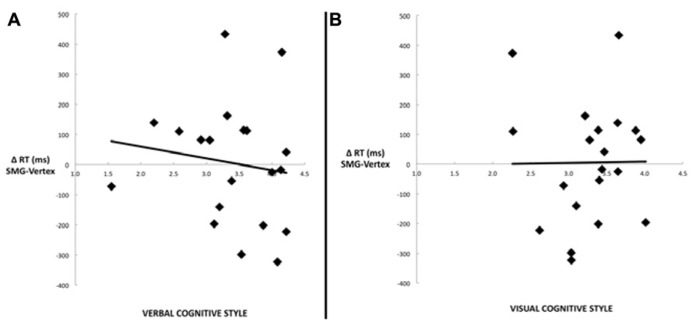
**Scatter plots depicting the lack of correlation between median response time differences (SMG–Vertex) for the picture–word condition as a result of left SMG rTMS and (A) the verbal cognitive style (*r* = –0.14, *p *= 0.56), or (B) the visual cognitive style (*r* = 0.01, *p *= 0.97)**.

Another commonly used method for assessing cognitive style is to calculate the difference score between verbal and visual dimensions of the cognitive style measures (Mayer and Massa, 2003; Massa and Mayer, 2006). **Figure 4** illustrates the correlation between the effect of rTMS and the verbal–visual cognitive style difference score (*r* = -0.46, *p *= 0.04). This correlation is in the hypothesized direction, but does not survive the Bonferroni correction. No significant correlation was found between cognitive style and median response time differences (SMG – Vertex) for any task condition (all *r* values < 0.30, all p values > 0.20; **Figure 5**).

## DISCUSSION

Previous neuroscience investigations have revealed that individual differences in neural activity reflect different strategies associated with visual and verbal cognitive styles, both when behavioral performance differs as a function of cognitive style (Hsu et al., 2011), and even when task performance does not differ (Gevins and Smith, 2000; Motes et al., 2008; Kraemer et al., 2009). In particular, results from our previous study (Kraemer et al., 2009), suggested the conversion hypothesis of verbal and visual cognitive styles, which posits that the verbal cognitive style correlates with the likelihood that one will use a verbal processing approach in order to complete a task, even when the stimuli appear as pictures. Here we present direct behavioral evidence in support of this hypothesis. Specifically, disrupting the activity of a functionally-defined word-responsive region of left SMG (and surrounding cortex) impaired performance on a task involving judgments about the properties of a visual stimulus held briefly in working memory and then compared to verbal descriptions of similar properties. Notably, the detrimental effect of rTMS on this task was predicted significantly by the verbal cognitive style, but not by the visual cognitive style. This pattern supports the interpretation given in the previous study that left SMG activity represents a verbal method of completing the task, which, when available, is as successful as the visual approach. But when this verbal processing mechanism is disrupted, individuals who are more likely to rely on it – defined here as those who have high scores on the verbal cognitive style scale – become impaired at a task on which performance (i.e., accuracy or response time) otherwise does not correlate with cognitive style. In particular, this effect was seen in the picture–word condition as hypothesized, given the similarity between this condition and the purported verbal labeling strategy (i.e., converting a picture into a verbal description). Notably, this correlation was observed despite no overall main effect of rTMS, further supporting the hypothesis that disrupting SMG function should only impair those individuals using the verbal strategy and not those who are using the equally effective visual strategy. Moreover, the fact that an rTMS-induced decline in accuracy correlating with the verbal cognitive style was observed without any change in response times may indicate that participants were not aware of the impairment to their normal strategy incurred by rTMS and did not try to engage in alternate compensatory processing or error-checking, either of which would likely have resulted in longer response times. We also expected to find a significant correlation between verbal cognitive style and rTMS-induced performance impairment in the picture–picture condition, but we did not observe such an effect. However, the behavioral results indicate that the picture–picture condition was by far the easiest task condition, with overall average accuracy of 98% and response times under 800 ms; therefore this null finding is difficult to interpret, as it may be due to ceiling effects. Likewise, similarly low error rates in the word–word and word–picture conditions prevent the current results from conclusively ruling out alternative hypotheses, such as meshing, that would have predicted a significant correlation between verbal cognitive style and rTMS-induced performance impairment in these two conditions. The conversion hypothesis also predicts that the visual cognitive style correlates with the likelihood that one will use a visual processing approach to complete verbal tasks, although this was not tested here due to the infeasibility of stimulating the corresponding region of fusiform cortex using TMS.

It is worth noting that the design approach used here, in which participants underwent a functional scanning protocol to localize the rTMS targets on an individual subject basis, likely facilitated our ability to see the observed behavioral effects. As seen in **Figure 2**, there was a great deal of variance across subjects in localizing the peak of activity in the left SMG for the given contrast of words greater than pictures. Had we not used this approach, but rather relied on previously-reported coordinates or anatomical landmarks, we might not have captured this variance and we might have instead found a null result. Thus, especially when using TMS to investigate effects that rely on individual differences in behavior, it may be critical to take such measures in order to achieve the highest sensitivity to individual differences in functional neuroanatomy. However, although each participant’s functional peak was localized to SMG, due to the likely spread of stimulation to nearby regions, we cannot infer that any behavioral effects observed by stimulation to these sites are due specifically to interference of SMG function and not, for example, to interference with STG function. Nonetheless, previous research has also associated STG with language production, language perception, and verbal working memory (for a review, see Buchsbaum et al., 2001; Price, 2010). Therefore, the present effects of rTMS seem confined to left peri-Sylvian regions that are associated with language and verbal working memory (Paulesu et al., 1993; Awh et al., 1996; Dronkers et al., 2004), and thus support our hypothesis regarding the verbal cognitive style.

Going forward with the study of cognitive styles, the present results speak to two relevant points, one methodological and one theoretical. On a methodological note, these findings indicate that interference with a task approach or strategy can be an effective design method for determining the relation between that approach and the cognitive style with which it is purportedly associated. In future rTMS studies, it would be beneficial to use tasks that are more difficult than the one used here, in order to reliably detect the effects of rTMS on error rates and response times for all task conditions. Although rTMS is not an available option for interfering with the function of many visual cortical areas that may be associated with the visual cognitive style, such as the previously noted fusiform region (Kraemer et al., 2009), perhaps a behavioral interference paradigm (e.g., overloading visual working memory with a dual task) would prove successful at accomplishing a comparable form of interference.

On a more theoretical note regarding the influence of cognitive styles on behavior, the fact that, absent interference from rTMS, cognitive style does not predict accuracy indicates that this task is part of a class of tasks for which either a verbal or a visual strategy is equally successful. This is not always the case, however, as some tasks may lend themselves more to one processing strategy than another. Some evidence supporting this distinction exists in the finding that verbal, but not visual, cognitive style predicted recall for the names of a set of previously-seen objects when they were presented in pictorial form, but not when they were presented as words (Constantinidou and Baker, 2002). Thus a strategy that includes the use of a verbal label may have been helpful in later recall of the names of pictured objects, a finding that is consistent with the conversion hypothesis as well as dual coding theory (Paivio, 1979), in which having a verbal and a visual code leads to enhanced memory relative to either code alone.

More generally, verbalization has been shown to enhance performance on visual tasks, such as categorization of visual stimuli (Lupyan et al., 2007; Lupyan and Thompson-Schill, 2012). In contrast, impaired verbal abilities, either through articulatory suppression (Baldo et al., 2005) or left-hemisphere lesion (Kertesz and McCabe, 1975; Baldo et al., 2005), have been associated with lower performance on visual reasoning tasks, such as Raven’s Matrices (Raven et al., 2000) and the Wisconsin Card Sorting Test (Heaton et al., 1993). The present findings suggest that the degree to which the verbalizing component affects performance on these visual tasks may differ across individuals as a function of verbal cognitive style. Conversely, research with high-functioning autistic populations (Soulières et al., 2009; Sahyoun et al., 2010) has provided examples of individuals who rely on a visual strategy to solve complex tasks, whereas control subjects depend on language processing for the same tasks. The recruitment of these visual strategies by autistic individuals is associated with strong visuospatial abilities (corresponding with intact and highly-activated visual association cortex) and weak language abilities (corresponding with impaired structure and function of language-related brain regions). The results of Kraemer et al. (2009) suggest that visual cognitive style may indicate a similar reliance on visual processing during language-based tasks in neurotypical populations.

As these examples demonstrate, it will be important to focus future research on which types of tasks afford success differentially for one strategy versus another, and what is the nature of the strategies used in these types of tasks. That is, there may not be a single verbal strategy or a single visual strategy for all tasks, but rather they may adapt to goal-oriented and stimulus-oriented constraints of each task situation. Describing the tasks and task strategies for which performance varies will be an important next step in characterizing the nature of cognitive styles and in determining their potential value in terms of learning and memory.

## Conflict of Interest Statement

The authors declare that the research was conducted in the absence of any commercial or financial relationships that could be construed as a potential conflict of interest.
